# 锁骨上超声检查提高原发性肺癌N分期的应用价值

**DOI:** 10.3779/j.issn.1009-3419.2014.09.04

**Published:** 2014-09-20

**Authors:** 钊 刘, 文 程, 鹏飞 李, 一欣 孙, 秋程 王

**Affiliations:** 1 150081 哈尔滨，哈尔滨医科大学附属肿瘤医院超声科 Department of Ultrasound, Harbin Medical University Cancer Hospital, Harbin 150081, China; 2 150081 哈尔滨，哈尔滨医科大学附属肿瘤医院影像诊断中心 Department of Diagnostic Radialogy, Harbin Medical University Cancer Hospital, Harbin 150081, China

**Keywords:** 肺肿瘤, 锁骨上淋巴结, 超声, 增强CT, 分期, 诊断, Lung neoplasms, Supraclavicular lymph node, Ultrasound, Enhanced CT, Staging, Diagnosis

## Abstract

**背景与目的:**

原发性肺癌是常见的恶性肿瘤之一，术前准确的局部淋巴结（N）分期可避免不必要的手术创伤，N3期已非手术治疗指征。本研究旨在探讨超声在诊断原发性肺癌锁骨上淋巴结转移及确定其分期中的应用价值。

**方法:**

回顾性分析2012年10月-2013年11月经病理确诊为肺癌患者131例，所有患者均在术前行锁骨上区域的超声和增强计算机断层扫描（computed tomography, CT），对检查结果为阳性的患者行超声引导下穿刺活检，将组织病理学诊断作为淋巴结转移的诊断标准，对比两种检查方法与病理结果的一致性。

**结果:**

131例肺癌患者中经病理证实共有50例为锁骨上淋巴结转移，经超声检查阳性者54例，转移者50例；增强CT检查阳性者41例，其中36例为恶性。超声的灵敏度、特异度、正确指数、阳性预测值及阴性预测值（分别为100%、95.06%、95.06%、92.59%、100%）明显高于增强CT（分别为72%、93.83%、65.83%、87.80%、84.44%）。两种方法在确定肺癌TNM分期准确性的差异存在统计学意义（*P* < 0.01）。

**结论:**

与增强CT相比，超声在原发性肺癌锁骨上淋巴结转移方面具有较高的准确性、敏感性和特异性，并且能够更加准确地确定原发性肺癌的TNM分期。

## Abstract (en)

**Background and objective:**

Primary lung cancer has been common malignant tumors. Accurate preoperative N staging can avoid unnecessary surgical operations, and patients with N3 has non-surgical treatment of indications. The aim of this study is to investigate the clinical value of ultrasound in the diagnosis of supraclavicular lymph node metastasis and staging of primary lung cancer.

**Methods:**

We retrospectively analyzed 131 patients who were pathologically diagnosed with lung cancer from October 2012 to November 2013. All patients received ultrasound and contrast-enhanced computed tomograohy (CT) examination of the supraclavicular area, and those who were with positive results underwent ultrasound-guided biopsy. The accuracy of the two methods was compared according to their consistency with the pathological results. The pathological diagnosis was used as the diagnosis standard of lymph node metastasis.

**Results:**

Tere were 50 cases of patients who were confrmed supraclavicular lymph node metastasis pathologically in a total of 131 cases of patients with lung cancer. Fify-four cases were tested positive by ultrasound, and 50 cases were pathologically proven to be with lymph node metastasis. For comparison, the ratio was 36 out of 41 for contrast-enhanced CT. The sensitivity, specifcity, You den's index, the positive predictive value and the negative predictive value of ultrasound (100%, 95.06%, 95.06%, 92.59%, 100%) was signifcantly higher than that of contrast-enhanced CT (72%, 93.83%, 65.83%, 87.80%, 84.44%). The differences of accuracy of the two methods in TNM staging of lung cancer had statistically signifcance (*P* < 0.01).

**Conclusion:**

Compared with enhanced CT, ultrasound examination has high accuracy, sensitivity and specifcity for primary supraclavicular lymph node metastasis in lung cancer, and at the same time can determine the TNM staging of primary lung cancer more accurately.

原发性肺癌是常见的恶性肿瘤之一，同时也是肿瘤死亡的主要病因，其治疗方案及预后首先取决于肿瘤的组织病理类型和临床分期^[1]^。锁骨上淋巴结转移是原发性肺癌常见的转移方式，根据肿瘤-淋巴结-转移(tumor-node-metastasis, TNM)分期分期标准，N3期已意味着不适宜手术^[2, 3]^。20世纪90年代彩色多普勒(color doppler flow imaging, CDFI)超声就已用于良恶性淋巴结的鉴别诊断^[4, 5]^。对于原发性肺癌患者，主要是行锁骨上超声和增强计算机断层扫描(computed tomography, CT) ^[6-8]^。同时，超声可以联合穿刺活检^[9-11]^取病理。本研究对经病理确诊为原发性肺癌的患者行锁骨上超声及增强CT，比较二者检测结果与病理结果的一致性，进而对比二者检查肺癌锁骨上淋巴结转移的价值，及两种方法检查前后原发性肺癌TNM分期的变化情况。

## 1 资料和方法

### 1.1 一般资料

2012年10月-2013年11月入组131例原发性肺癌患者，其中男性68例，年龄37岁-77岁，平均年龄56.8岁；女性63例，年龄33岁-76岁，平均年龄58.8岁。对入组患者行锁骨上超声之前，经胸部增强CT所示且由临床医生进行分期，包括Ⅰa期6例、Ⅰb期5例、Ⅱa期6例、Ⅱb期5例、Ⅲa期60例、Ⅲb期14例、Ⅳ期35例，其中原发肿瘤T：T1a 11例、T1b 41例、T2a 45例、T2b 12例、T3 13例、T4 9例；区域淋巴结N：N0 21例、N1 7例、N2 80例、N3 23例；远处转移M：M0 96例，M1共35例，其中M1a 4例，均为肺内播散，M1b 24例包括骨转移10例、左侧或(和)右侧肾上腺区转移3例、脑转移8例、肝转移3例，同时发生M1a和M1b共7例。所有患者包括鳞癌35例，腺癌72例，小细胞癌24例。

### 1.2 方法

#### 1.2.1 CT检测

使用Philips brilliance-64多层螺旋CT机。扫描范围：下颈部、锁骨上区及胸部。扫描层厚5 mm。使用高压注射器，经肘静脉注入非离子型造影剂(优维显370) 100 mL，延迟时间40 s-50 s，注入速率3.5 mL/s-4.0 mL/s。锁骨上淋巴结肿大标准：短径(S)≥5 mm。采用双盲法，由两位高年资放射科医师分别阅片。二者意见不统一时，由第三名高年资放射科医师再次阅片，最后综合三者结果。

#### 1.2.2 超声检测与穿刺方法

锁骨上超声与增强CT之间间隔不超过1周。采用SIEMENS Sequoia 512超声仪器，常规检测锁骨上淋巴结(图 1)。二维超声检查锁骨上淋巴结肿大的标准为其短径(S)≥5 mm，纵横比：长径(L) /短径(S) < 2。

**1:**
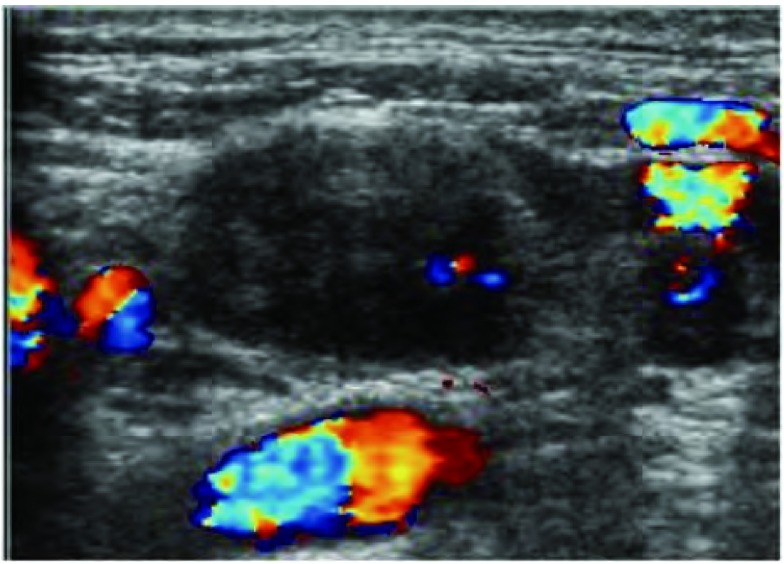
锁骨上转移性淋巴结在声像图中表现为低回声结节，皮髓质分界不清，内部及周边可见血流信号。 The ultrasound image performance of supraclavicular metastasis lymph nodes were hypoechoic nodules with cortex and medulla unclear boundaries and internal and peripheral blood flow signals.

穿刺操作过程由经验丰富的医生完成，患者取仰卧位，以2%的盐酸利多卡因局部麻醉，经超声引导以16 G活检针进针至肿大淋巴结边缘(图 2)，取出3条组织，以10%福尔马林固定送至病理科，行组织病理学诊断。

**2:**
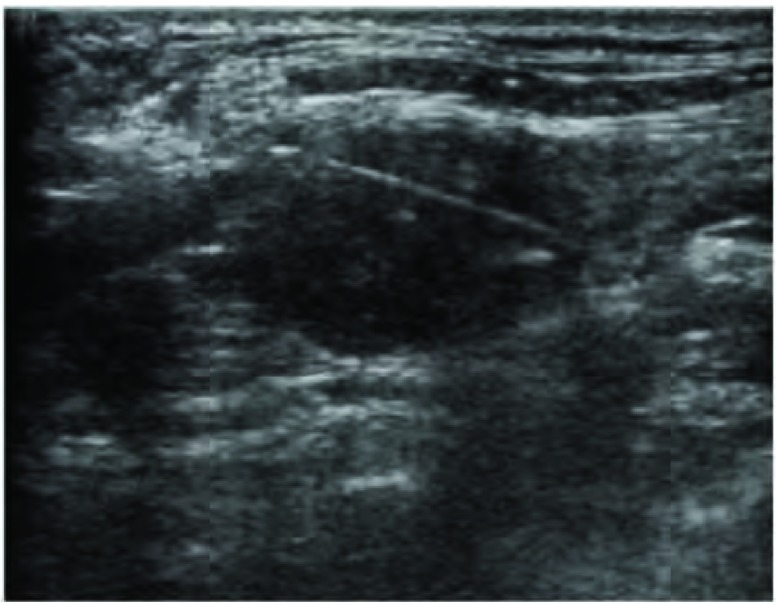
超声引导下穿刺活检，肿大的锁骨上淋巴结内可见清晰的穿刺针道。 Puncture biopsy guided by ultrasound. We can see needle path in the supraclavicular lymph node clearly.

#### 1.2.3 结果分析方法

以组织病理学结果作为参考标准，分析并比较超声检查和增强CT检查锁骨上淋巴结的结果，得出结论。

### 1.3 统计学方法

应用SPSS 16.0软件进行统计分析，计数资料采用卡方检验，以*P* < 0.05为差异具有统计学意义。

## 2 结果

### 2.1 超声与增强CT诊断结果的比较

131例患者经病理结果证实共有50例(鳞癌10例、腺癌27例、小细胞癌13例)发生锁骨上淋巴结转移。超声与增强CT对锁骨上淋巴结诊断结果的比较见表 1。两种检查方法诊断效能的对比结果见表 2。因此，在检查原发性肺癌锁骨上淋巴结方面，超声要明显优于增强CT。

**1:** 超声与增强CT检查锁骨上淋巴结的诊断结果 Diagnosis results of ultrasound and contrast-enhanced CT examination of supraclavicular lymph nodes

Examination	Pathologic results by ultrasound-guided biopsy		Total
	Malignant	Benign	
Ultrasound			
Positive	50	4	54
Negative	0	77	77
Contrast-enhanced CT			
Positive	36	5	41
Negative	14	76	90
Total	50	81	131
CT: computed tomography.			

**2:** 超声和增强CT的诊断效能 The diagnostic effect of ultrasound and contrast-enhanced CT

Index	Examination	
	Ultrasound	Contrast-enhanced CT
Sensitivity	100%	72%
Specificity	95.06%	93.83%
Youden's index	95.06%	65.83%
Positive predictive value	92.59%	87.80%
Negative predictive value	100%	84.44%
Sensitivity=number of true positive/(number of true positive+number of false negative)×100%; Specificity=number of true negative/(number of true negative+number of false positive)×100%; Youden's index=(Sensitivity+Specificity)-1; Positive predictive value=number of true positive/(number of true positive+number of false positive)×100%; Negative predictive value=number of true negative/(number of true negative+number of false negative)×100%.		

### 2.2 超声与增强CT对TNM分期影响的比较

50例患者发生锁骨上淋巴结转移(即N3)，导致其TNM分期也发生相应的改变(升为Ⅲb期)，但部分患者已经处于Ⅲb期或存在远处转移(Ⅳ期)，其TNM分期并不发生改变。经超声诊断阳性者54例中共有23例TNM分期发生改变(Ⅰa期2例、Ⅰb期2例、Ⅱa期2例、Ⅱb期4例，Ⅲa期13例)，而经锁骨上区域增强CT诊断的41例阳性患者中有9例TNM分期发生改变(Ⅰb期1例、Ⅱa期1例、Ⅱb期1例，Ⅲa期6例)。二者经卡方检验证实存在明显差异(*χ*^2^=6.977，*P* < 0.01，表 3)，说明在确定肺癌分期方面与增强CT相比，超声具有较高的价值。

**3:** 比较超声与增强CT对肺癌TNM分期的影响 Comparing the influence of ultrasound and contrast-enhanced CT of lung cancer TNM staging

Examination	TNM staging changed	TNM staging unchanged	Total
Ultrasound	23	108	131
Contrast-enhanced CT	9	122	131
Total	32	230	262
TNM: tumor-node-metastasis.			

50例患者包括N0期12例、N1期2例、N2期24例、N3期12例(经胸部增强CT确定的原N分期)；同时，其中22例远处转移，包括M1a 1例，M1b 17例，同时发生M1a和M1b 4例。M1期与M0期之间存在明显差异(*χ*^2^=12.335, *P* < 0.01)，说明M1期患者更容易发生锁骨上淋巴结转移；同理，N 2期患者也较易发生锁骨上淋巴结转移(*χ*^2^=5.809, *P* < 0.05)(表 4)。

**4:** N2和M分期与锁骨上淋巴结转移的关系 The relationship between N2/M staging and supraclavicular lymph node metastasis

N2/M staging	Supraclavicular lymph node metastasis was confirmed by pathology		Total
	Malignant	Benign	
N2	24	56	80
Non-N2	26	25	51
M1	22	13	35
M0	28	68	96
Total	50	81	131
Non-N2 includes N0、N1、N3.			

原发性肺癌锁骨上淋巴结转移与原发肿瘤T分期的关系如表 5，其*Pearson*相关系数*γ*=0.204，*P*=0.019，说明T分期与锁骨上淋巴结转移具有弱相关性。

**5:** 原发性肺癌T分期与锁骨上淋巴结转移的关系 The relationship between T staging of primary lung cancer and supraclavicular lymph node metastasis

Supraclavicular lymph node	T staging of primary lung cancer						Total
	T1a	T1b	T2a	T2b	T3	T4	
Positive	2	13	17	6	7	5	50
Negative	9	28	28	6	6	4	81
Total	11	41	45	12	13	9	131

## 3 讨论

Ⅲb期原发性肺癌患者已不适宜手术治疗^[12]^。根据2009年国际抗癌联盟(Union for International Cancer Control, UICC)公布的修订后的第七版肺癌分期，锁骨上淋巴结转移属于N3期，在TNM分期中为Ⅲb期(任何TN3M0、T4N2M0)。因此，不论T分期如何，只要经病理结果证实为锁骨上淋巴结转移时，即为Ⅲb期，已不适宜手术^[3]^。锁骨上淋巴结转移的检出对原发性肺癌的进一步治疗非常重要，可避免一些不必要的侵入性检查和治疗。有研究^[6, 13, 14]^表明超声和CT检查明显优于触诊，近年来提倡使用超声和增强CT作为常规检查，以提高原发性肺癌患者锁骨上淋巴结转移的检出率。

相关文献显示其他恶性肿瘤患者，如食管癌^[15]^、黑色素瘤^[16]^、肺癌^[10, 11]^患者锁骨上淋巴结检查中，超声及超声引导下穿刺活检组织学分析具有明显的优越性，因此已在临床上得到广泛应用。

本研究中，超声检查阳性者有50例经病理证实为转移淋巴结，其中14例增强CT并未检测出。因此，在检查锁骨上淋巴结转移方面，超声的诊断效能要优于增强CT，这与Hoosein等^[9]^研究结果相近。与增强CT相比，超声检查在确定原发性肺癌N分期方面要更加准确。本研究中经增强CT检查改变的患者中不包括Ia期，可能是由于样本量较小所致。

Van Overhagen等^[6]^研究结果显示，原发性肺癌锁骨上淋巴结是否转移与T分期无相关性，而本研究中显示两者存在弱相关性，即较高期别的T分期易发生锁骨上淋巴结转移。本研究显示发生远处转移的患者常伴有锁骨上淋巴结转移，与Van Overhagen等^[6]^研究结果相同。另外本研究显示N2患者常伴有锁骨上淋巴结转移，而Van Overhagen等^[6]^认为N3患者常发生锁骨上淋巴结转移。且总体来说，M1者较N2者相比更易伴有锁骨上淋巴结转移。

组织病理学结果是诊断肺癌锁骨上淋巴结转移的"金标准"。超声引导下穿刺活检是一项简单、安全且创伤小的检查手段^[11, 17]^。与增强CT相比，超声操作流程更简单，而且可以联合超声引导下穿刺活检，有效地诊断肺癌锁骨上淋巴结是否发生转移，尤其是对触诊未检出的淋巴结，因此临床上偶尔会出现TNM分期“跳跃”的现象。

但是，本研究仍存在着一定的局限性。首先，由于外科扩大根治手术清扫的淋巴结并不包括锁骨上淋巴结，部分已手术的患者中可能存在锁骨上淋巴结转移，因此超声和增强CT的实际敏感度应该低于我们报道的数据。

其次，本研究采用穿刺活检病理结果作为原发性肺癌患者锁骨上淋巴结转移的金标准。由于操作者的穿刺手法及熟练程度不同、穿刺位置不够准确而取得坏死组织、肿瘤细胞被掩盖或肿瘤异质性等原因，均有可能导致出现假阴性的病理结果。

综上所述，增强CT适用于原发肿瘤、肺门及纵隔淋巴结及远处转移的检测，而超声及超声引导下穿刺活检在原发性肺癌锁骨上淋巴结是否发生转移方面具有较高的应用价值，因此联合应用增强CT及锁骨上区域超声检查和超声引导下穿刺活检，有助于确定原发性肺癌TNM分期和制定治疗方案。

## References

[1] Su MG, Li L (2012). Correlation between regional node metastasis and imageological characteristics in non-small cell lung cancer. Zhongguo Fei Ai Za Zhi.

[2] Mirsadraee S, Oswal D, Alizadeh Y (2012). The 7^th^ lung cancer TNM classification and staging system: Review of the changes and implications. World J Radiol.

[3] Ramnath N, Dilling TJ, Harris LJ (2013). Treatment of stage Ⅲ non-small cell lung cancer: diagnosis and management of lung cancer, 3^rd^ ed: American College of Chest Physicians evidence-based clinical practice guidelines. Chest.

[4] Taniyama Y, Nakamura T, Mitamura A (2013). A strategy for supraclavicular lymph node dissection using recurrent laryngeal nerve lymph node status in thoracic esophageal squamous cell carcinoma. Ann Thorac Surg.

[5] Asakura H, Ohtsuka M, Ito H (2005). Long-term survival after extended surgical resection of intrahepatic cholangiocarcinoma with extensive lymph node metastasis. Hepatogastroenterology.

[6] Van Overhagen H, Brakel K, Heijenbrok MW (2004). Metastases in supraclavicular lymph nodes in lung cancer: assessment with palpation, US, and CT. Radiology.

[7] Fultz PJ, Feins RH, Strang JG (2002). Detection and diagnosis of nonpalpable supraclavicular lymph nodes in lung cancer at CT and US. Radiology.

[8] Ryu EB, Oh KS, Jeong KS (2012). Supraclavicular lymph node metastasis from various malignancies:assessment with ^18^F-fluorodeoxyglucose positron emission tomography/CT, contrast-enhanced CT and ultrasound. J Korean Soc Radiol.

[9] Hoosein MM, Barnes D, Khan AN (2011). The importance of ultrasound in staging and gaining a pathological diagnosis in patients with lung cancer-a two year single centre experience. Thorax.

[10] Ozkan G, Tutar M, Bayram M (2009). The impact of ultrasonography-guided fine needle aspiration of no palpable supraclavicular lymph nodes on diagnosis and staging in advanced lung cancer. Tuberk Toraks.

[11] Kendirlinan R, Ozkan G, Bayram M (2011). Ultrasound guided fine-needle aspiration biopsy of metastases in nonpalpable supraclavicular lymph nodes in lung cancer patients. Multidiscip Respir Med.

[12] Russell K, Healy B, Pantarotto J (2014). Pronostic factors in the radical nonsurgical treatment of stage ⅢB non-small-cell lung cancer. Clin Lung Cancer.

[13] Dionisios Spyratos, Diamantis Chloros, Ariadni Mandrali (2011). FNA of palpable supraclavicular lymph nodes in lung cancer: comparison between palpation and ultrasound. Eur Respir J.

[14] Guan XX, Fu ZY, Wang SH (2012). The clinical value of color doppler ultrasound on supraclavicular lymph node metastasis in primary lung cancer. Shi Yong Ai Zheng Za Zhi.

[15] Guilbert AS, Xavier L, Ammouche C (2013). Supraclavicular ultrasound-guided catheterization of the subclavian vein in pediatric and neonatal ICUs: a feasibility study. Pediatr Crit Care Med.

[16] Blum A, Schlagenhauff B, Stroebel W (2000). Ultrasound examination of regional lymph nodes significantly improves early detection of locoregional metastases during the follow-up of patients with cutaneous melanoma: results of a prospective study of 1, 288 patients. Cancer.

[17] Kumaran M1, Benamore RE, Vaidhyanath R (2005). Ultrasound guided cytological aspiration of supraclavicular lymph nodes in patients with suspected lung cancer. Thorax.

